# 2-(1*H*-Benzimidazol-2-yl)-4,6-dichloro­phenol

**DOI:** 10.1107/S1600536810027844

**Published:** 2010-07-17

**Authors:** Li-Lu Han

**Affiliations:** a Hunan Yongzhou Vocational College, Yongzhou Hunan 425100, People’s Republic of China

## Abstract

The title compound, C_13_H_8_Cl_2_N_2_O, was prepared by the reaction of 3,5-dichloro-2-hy­droxy­benzaldehyde with 1,2-diamino­benzene in methanol at ambient temperature. The title mol­ecule is essentially planar, the mean deviation from the plane of the non-H atoms being 0.037 (2) Å. There is an intra­molecular O—H⋯N hydrogen bond in the mol­ecule. In the crystal, symmetry-related mol­ecules are linked through N—H⋯O hydrogen bonds, forming polymeric chains propagating in [001]. The chains are linked by π–π inter­actions involving the dichloro­phenol ring and the benzoimidazole ring system [centroid–centroid distances = 3.535 (2) and 3.724 (2) Å].

## Related literature

For the preparation and crystal structures of some Schiff bases bearing a C=N double bond, see: Jeseentharani *et al.* (2010 ▶); Hamaker *et al.* (2010 ▶); Tanaka *et al.* (2010 ▶); Tunç *et al.* (2009 ▶); Khalaji *et al.* (2010 ▶). For standard bond distances, see: Allen *et al.* (1987 ▶).
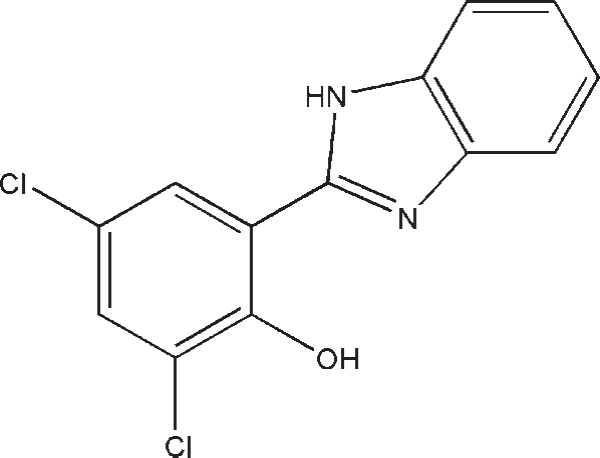


## Experimental

### 

#### Crystal data

C_13_H_8_Cl_2_N_2_O
*M* *_r_* = 279.11
Monoclinic, 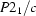

*a* = 11.850 (3) Å
*b* = 7.446 (3) Å
*c* = 13.947 (2) Å
β = 104.261 (3)°
*V* = 1192.7 (6) Å^3^
*Z* = 4
Mo *K*α radiation
μ = 0.53 mm^−1^
*T* = 298 K
0.21 × 0.20 × 0.18 mm

#### Data collection

Bruker SMART CCD area-detector diffractometer
Absorption correction: multi-scan (*SADABS*; Bruker, 2001 ▶) *T*_min_ = 0.897, *T*_max_ = 0.911
6117 measured reflections
2562 independent reflections
1810 reflections with *I* > 2σ(*I*)
*R*_int_ = 0.035

#### Refinement

*R*[*F* ^2^ > 2σ(*F* ^2^)] = 0.042
*wR*(*F* ^2^) = 0.127
*S* = 1.03
2562 reflections
167 parameters
1 restraint
H atoms treated by a mixture of independent and constrained refinement
Δρ_max_ = 0.26 e Å^−3^
Δρ_min_ = −0.23 e Å^−3^

### 

Data collection: *SMART* (Bruker, 2007 ▶); cell refinement: *SAINT* (Bruker, 2007 ▶); data reduction: *SAINT*; program(s) used to solve structure: *SHELXS97* (Sheldrick, 2008 ▶); program(s) used to refine structure: *SHELXL97* (Sheldrick, 2008 ▶); molecular graphics: *SHELXTL* (Sheldrick, 2008 ▶); software used to prepare material for publication: *SHELXTL*.

## Supplementary Material

Crystal structure: contains datablocks global, I. DOI: 10.1107/S1600536810027844/su2193sup1.cif

Structure factors: contains datablocks I. DOI: 10.1107/S1600536810027844/su2193Isup2.hkl

Additional supplementary materials:  crystallographic information; 3D view; checkCIF report

## Figures and Tables

**Table 1:** Hydrogen-bond geometry (Å, °)

*D*—H⋯*A*	*D*—H	H⋯*A*	*D*⋯*A*	*D*—H⋯*A*
O1—H1⋯N2	0.82	1.85	2.582 (2)	148
N1—H1*A*⋯O1^i^	0.90 (3)	2.39 (2)	3.145 (2)	143 (3)

Symmetry code: (i) 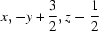.

**Fig. 1.**
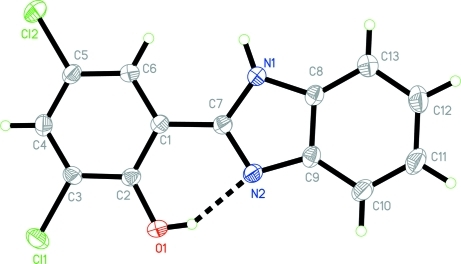
The molecular structure of the title compound. Displacement ellipsoids are drawn at the 30% probability level. The intramolecular O-H···N hydrogen bond is shown as a dashed line.

**Fig. 2.**
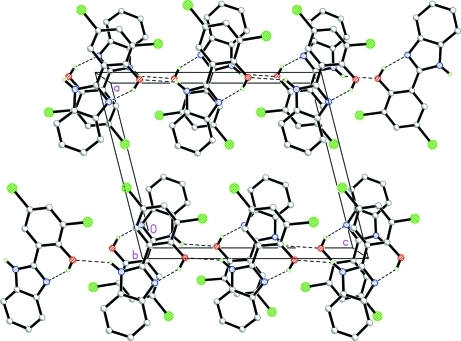
The crystal packing of the title compound, viewed along the b-axis. The O-H···N and N-H···O hydrogen bonds are shown as dashed lines (see Table 1 for details).

## supplementary crystallographic information

### Comment

The condensation reaction of aldehydes with primary amines readily leads to the formation of Schiff bases bearing a C=N double bond (Jeseentharani *et al.*, 2010; Hamaker *et al.*, 2010; Tanaka *et al.*, 2010; Tunç *et al.*, 2009; Khalaji *et al.*, 2010). Herein, we report on the structure of the title compound, the unexpected result of the Schiff base condensation reaction of 3,5-dichloro-2-hydroxybenzaldehyde with 1,2-diaminobenzene.

The title molecule (Fig. 1)  is essentially planar, with the mean deviation from the plane of all the non-H atoms being 0.037 (2) Å. There is an intramolecular O—H···N hydrogen bond (Table 1) in the molecule, as shown in Fig. 1. All the bond lengths are within normal ranges (Allen *et al.*, 1987).

In the crystal symmetry related molecules are linked through an intermolecular N—H···O hydrogen bond to form polymer chains propagating in [001] (Table 1 and Fig. 2). These chains are linked via π–π stacking interactions involving rings N1/N2/C7-C9 and C1-C6 [symmetry operation: 2-x, 2-y, 1-y], with a centroid-to-centroid distance of 3.535 (2) Å, and rings C1-C6 and C8-C13 [symmetry code: 2-x, 1-y, 1-z], with a centroid-to-centroid distance of 3.724 (2)Å.

### Experimental

3,5-Dichloro-2-hydroxybenzaldehyde (1 mmol, 0.19 g) and 1,2-diaminobenzene (1 mmol, 0.11 g) were dissolved in methanol (30 ml). The mixture was stirred for 30 mins. at RT to give a yellow solution. Yellow single crystals were obtained by slow evaporation of the solution in air.

### Refinement

Atom H1A was located in a difference Fourier map and its positional parameters were refined with a fixed isotropic thermal parameter of 0.08 Å^2^. The remaining H-atoms were positioned geometrically and refined as riding: C—H = 0.93 Å, O—H = 0.82 Å, with *U*_iso_(H) = 1.2*U*_eq_(C) and 1.5*U*_eq_(O).

### Crystal data

**Table:**

C_13_H_8_Cl_2_N_2_O	*F*(000) = 568
*M**_r_* = 279.11	*D*_x_ = 1.554 Mg m^−^^3^
Monoclinic, *P*2_1_/*c*	Mo *K*α radiation, λ = 0.71073 Å
Hall symbol: -P 2ybc	Cell parameters from 1560 reflections
*a* = 11.850 (3) Å	θ = 3.0–26.2°
*b* = 7.446 (3) Å	µ = 0.53 mm^−^^1^
*c* = 13.947 (2) Å	*T* = 298 K
β = 104.261 (3)°	Block, yellow
*V* = 1192.7 (6) Å^3^	0.21 × 0.20 × 0.18 mm
*Z* = 4	

### Data collection

**Table:**

Bruker SMART CCD area-detector diffractometer	2562 independent reflections
Radiation source: fine-focus sealed tube	1810 reflections with *I* > 2σ(*I*)
graphite	*R*_int_ = 0.035
ω scans	θ_max_ = 27.0°, θ_min_ = 3.0°
Absorption correction: multi-scan (*SADABS*; Bruker, 2001)	*h* = −15→12
*T*_min_ = 0.897, *T*_max_ = 0.911	*k* = −9→9
6117 measured reflections	*l* = −12→17

### Refinement

**Table:**

Refinement on *F*^2^	Primary atom site location: structure-invariant direct methods
Least-squares matrix: full	Secondary atom site location: difference Fourier map
*R*[*F*^2^ > 2σ(*F*^2^)] = 0.042	Hydrogen site location: inferred from neighbouring sites
*wR*(*F*^2^) = 0.127	H atoms treated by a mixture of independent and constrained refinement
*S* = 1.03	*w* = 1/[σ^2^(*F*_o_^2^) + (0.0678*P*)^2^] where *P* = (*F*_o_^2^ + 2*F*_c_^2^)/3
2562 reflections	(Δ/σ)_max_ = 0.001
167 parameters	Δρ_max_ = 0.26 e Å^−^^3^
1 restraint	Δρ_min_ = −0.23 e Å^−^^3^

### Special details

**Table:**

Geometry. All e.s.d.'s (except the e.s.d. in the dihedral angle between two l.s. planes) are estimated using the full covariance matrix. The cell e.s.d.'s are taken into account individually in the estimation of e.s.d.'s in distances, angles and torsion angles; correlations between e.s.d.'s in cell parameters are only used when they are defined by crystal symmetry. An approximate (isotropic) treatment of cell e.s.d.'s is used for estimating e.s.d.'s involving l.s. planes.
Refinement. Refinement of *F*^2^ against ALL reflections. The weighted *R*-factor *wR* and goodness of fit *S* are based on *F*^2^, conventional *R*-factors *R* are based on *F*, with *F* set to zero for negative *F*^2^. The threshold expression of *F*^2^ > σ(*F*^2^) is used only for calculating *R*-factors(gt) *etc*. and is not relevant to the choice of reflections for refinement. *R*-factors based on *F*^2^ are statistically about twice as large as those based on *F*, and *R*- factors based on ALL data will be even larger.

### Fractional atomic coordinates and isotropic or equivalent isotropic displacement parameters (Å^2^)

**Table:**

	*x*	*y*	*z*	*U*_iso_*/*U*_eq_	
Cl1	1.19596 (6)	0.99614 (9)	0.79805 (4)	0.0505 (2)	
Cl2	1.37522 (5)	0.87011 (11)	0.48582 (5)	0.0602 (3)	
N1	0.91704 (15)	0.6598 (3)	0.37972 (14)	0.0350 (4)	
N2	0.85158 (15)	0.7146 (3)	0.51409 (13)	0.0361 (4)	
O1	0.98220 (14)	0.8474 (2)	0.67436 (12)	0.0469 (4)	
H1	0.9240	0.8028	0.6378	0.070*	
C1	1.05692 (18)	0.7881 (3)	0.52982 (15)	0.0312 (5)	
C2	1.06961 (18)	0.8491 (3)	0.62790 (16)	0.0337 (5)	
C3	1.1788 (2)	0.9158 (3)	0.67846 (15)	0.0345 (5)	
C4	1.27187 (19)	0.9217 (3)	0.63599 (16)	0.0389 (5)	
H4	1.3434	0.9663	0.6713	0.047*	
C5	1.25768 (19)	0.8605 (3)	0.53975 (17)	0.0376 (5)	
C6	1.15119 (19)	0.7967 (3)	0.48692 (16)	0.0360 (5)	
H6	1.1423	0.7590	0.4219	0.043*	
C7	0.94248 (18)	0.7206 (3)	0.47531 (15)	0.0328 (5)	
C8	0.79936 (19)	0.6132 (3)	0.35518 (16)	0.0352 (5)	
C9	0.75956 (18)	0.6484 (3)	0.44004 (17)	0.0365 (5)	
C10	0.6424 (2)	0.6198 (4)	0.43936 (19)	0.0489 (7)	
H10	0.6147	0.6440	0.4949	0.059*	
C11	0.5702 (2)	0.5549 (4)	0.3542 (2)	0.0584 (8)	
H11	0.4922	0.5346	0.3520	0.070*	
C12	0.6117 (2)	0.5182 (4)	0.2698 (2)	0.0596 (8)	
H12	0.5604	0.4730	0.2135	0.071*	
C13	0.7263 (2)	0.5474 (3)	0.26834 (19)	0.0485 (6)	
H13	0.7532	0.5244	0.2123	0.058*	
H1A	0.968 (2)	0.649 (4)	0.342 (2)	0.080*	

### Atomic displacement parameters (Å^2^)

**Table:**

	*U*^11^	*U*^22^	*U*^33^	*U*^12^	*U*^13^	*U*^23^
Cl1	0.0599 (5)	0.0604 (4)	0.0299 (3)	−0.0096 (3)	0.0084 (3)	−0.0027 (3)
Cl2	0.0373 (4)	0.0907 (6)	0.0589 (4)	−0.0105 (3)	0.0236 (3)	−0.0014 (4)
N1	0.0306 (10)	0.0409 (11)	0.0340 (10)	−0.0020 (8)	0.0090 (8)	−0.0018 (8)
N2	0.0306 (10)	0.0451 (12)	0.0341 (10)	0.0002 (9)	0.0106 (8)	0.0037 (8)
O1	0.0365 (9)	0.0717 (13)	0.0367 (9)	−0.0072 (8)	0.0171 (7)	−0.0077 (8)
C1	0.0297 (11)	0.0315 (12)	0.0326 (11)	0.0013 (9)	0.0085 (9)	0.0032 (9)
C2	0.0327 (12)	0.0374 (13)	0.0326 (11)	0.0040 (9)	0.0112 (9)	0.0065 (9)
C3	0.0384 (13)	0.0367 (13)	0.0283 (11)	0.0003 (10)	0.0077 (9)	0.0038 (9)
C4	0.0318 (13)	0.0438 (14)	0.0384 (12)	−0.0037 (10)	0.0034 (10)	0.0046 (10)
C5	0.0303 (12)	0.0433 (14)	0.0409 (13)	0.0004 (10)	0.0118 (10)	0.0048 (10)
C6	0.0349 (12)	0.0411 (14)	0.0338 (11)	−0.0007 (10)	0.0123 (10)	−0.0020 (10)
C7	0.0331 (12)	0.0339 (12)	0.0320 (11)	0.0028 (10)	0.0088 (9)	0.0043 (9)
C8	0.0299 (12)	0.0370 (13)	0.0382 (12)	0.0004 (9)	0.0073 (9)	0.0048 (10)
C9	0.0281 (12)	0.0399 (13)	0.0402 (12)	0.0007 (10)	0.0058 (10)	0.0074 (10)
C10	0.0343 (13)	0.0657 (18)	0.0486 (15)	−0.0014 (12)	0.0138 (11)	0.0071 (12)
C11	0.0297 (13)	0.082 (2)	0.0626 (18)	−0.0054 (14)	0.0087 (13)	0.0066 (15)
C12	0.0426 (16)	0.075 (2)	0.0530 (16)	−0.0102 (14)	−0.0035 (13)	−0.0045 (14)
C13	0.0425 (15)	0.0583 (17)	0.0435 (14)	−0.0053 (13)	0.0082 (11)	−0.0039 (12)

### Geometric parameters (Å, °)

**Table:**

Cl1—C3	1.736 (2)	C4—C5	1.388 (3)
Cl2—C5	1.740 (2)	C4—H4	0.9300
N1—C7	1.370 (3)	C5—C6	1.379 (3)
N1—C8	1.395 (3)	C6—H6	0.9300
N1—H1A	0.90 (3)	C8—C13	1.393 (3)
N2—C7	1.320 (3)	C8—C9	1.403 (3)
N2—C9	1.394 (3)	C9—C10	1.402 (3)
O1—C2	1.350 (2)	C10—C11	1.369 (4)
O1—H1	0.8200	C10—H10	0.9300
C1—C6	1.393 (3)	C11—C12	1.409 (4)
C1—C2	1.414 (3)	C11—H11	0.9300
C1—C7	1.470 (3)	C12—C13	1.380 (4)
C2—C3	1.403 (3)	C12—H12	0.9300
C3—C4	1.375 (3)	C13—H13	0.9300
			
C7—N1—C8	106.74 (18)	C1—C6—H6	119.6
C7—N1—H1A	125 (2)	N2—C7—N1	112.43 (19)
C8—N1—H1A	128 (2)	N2—C7—C1	122.80 (19)
C7—N2—C9	106.08 (18)	N1—C7—C1	124.77 (19)
C2—O1—H1	109.5	C13—C8—N1	132.1 (2)
C6—C1—C2	119.70 (19)	C13—C8—C9	122.2 (2)
C6—C1—C7	121.93 (19)	N1—C8—C9	105.67 (19)
C2—C1—C7	118.35 (19)	N2—C9—C10	130.6 (2)
O1—C2—C3	118.9 (2)	N2—C9—C8	109.08 (19)
O1—C2—C1	123.35 (19)	C10—C9—C8	120.3 (2)
C3—C2—C1	117.71 (19)	C11—C10—C9	117.7 (2)
C4—C3—C2	122.2 (2)	C11—C10—H10	121.1
C4—C3—Cl1	119.11 (17)	C9—C10—H10	121.1
C2—C3—Cl1	118.67 (17)	C10—C11—C12	121.4 (2)
C3—C4—C5	119.1 (2)	C10—C11—H11	119.3
C3—C4—H4	120.4	C12—C11—H11	119.3
C5—C4—H4	120.4	C13—C12—C11	121.9 (2)
C6—C5—C4	120.4 (2)	C13—C12—H12	119.0
C6—C5—Cl2	120.51 (18)	C11—C12—H12	119.0
C4—C5—Cl2	119.04 (17)	C12—C13—C8	116.5 (2)
C5—C6—C1	120.8 (2)	C12—C13—H13	121.8
C5—C6—H6	119.6	C8—C13—H13	121.8

### Hydrogen-bond geometry (Å, °)

**Table:**

*D*—H···*A*	*D*—H	H···*A*	*D*···*A*	*D*—H···*A*
O1—H1···N2	0.82	1.85	2.582 (2)	148
N1—H1A···O1^i^	0.90 (3)	2.39 (2)	3.145 (2)	143 (3)

Symmetry codes: (i) *x*, −*y*+3/2, *z*−1/2.
